# Supplementary material for: Abrupt high-latitude climate events and decoupled seasonal trends during the Eemian
Source: Nat Commun. 2018 Jul 20;9:2851. doi: 10.1038/s41467-018-05314-1 (PMC6054633; doi:10.1038/s41467-018-05314-1)
Supplement: Supplementary file 1 — Supplementary Information [file 41467_2018_5314_MOESM1_ESM.pdf]

Supplementary information

Abrupt high-latitude climate events and decoupled seasonal trends during the Eemian

Salonen et al.

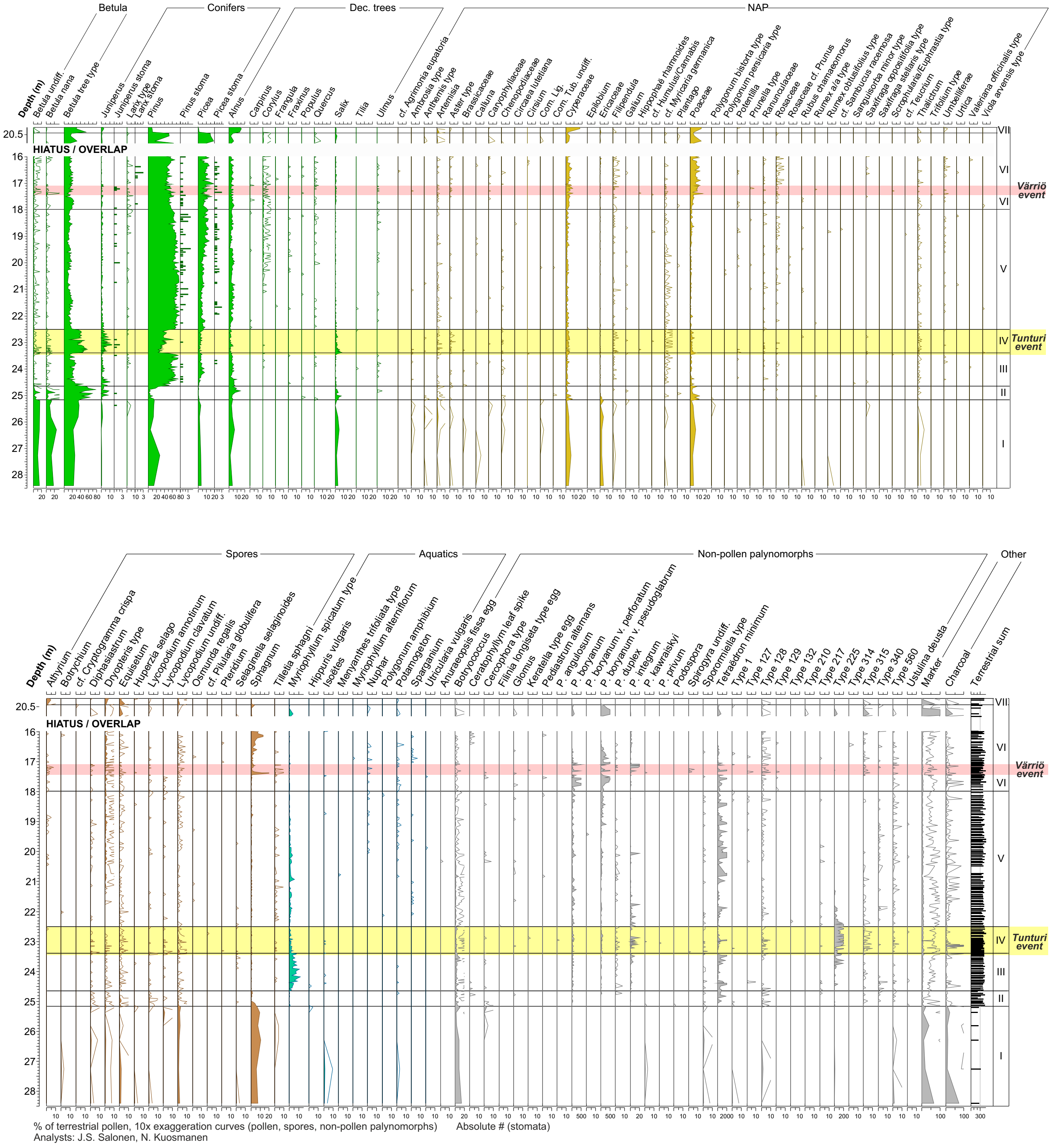

Supplementary Figure 1. Full pollen data of the Sokli Eemian sequence. The parallel core on the top of the sequence is shown with a 3x exaggeration of the depth axis.

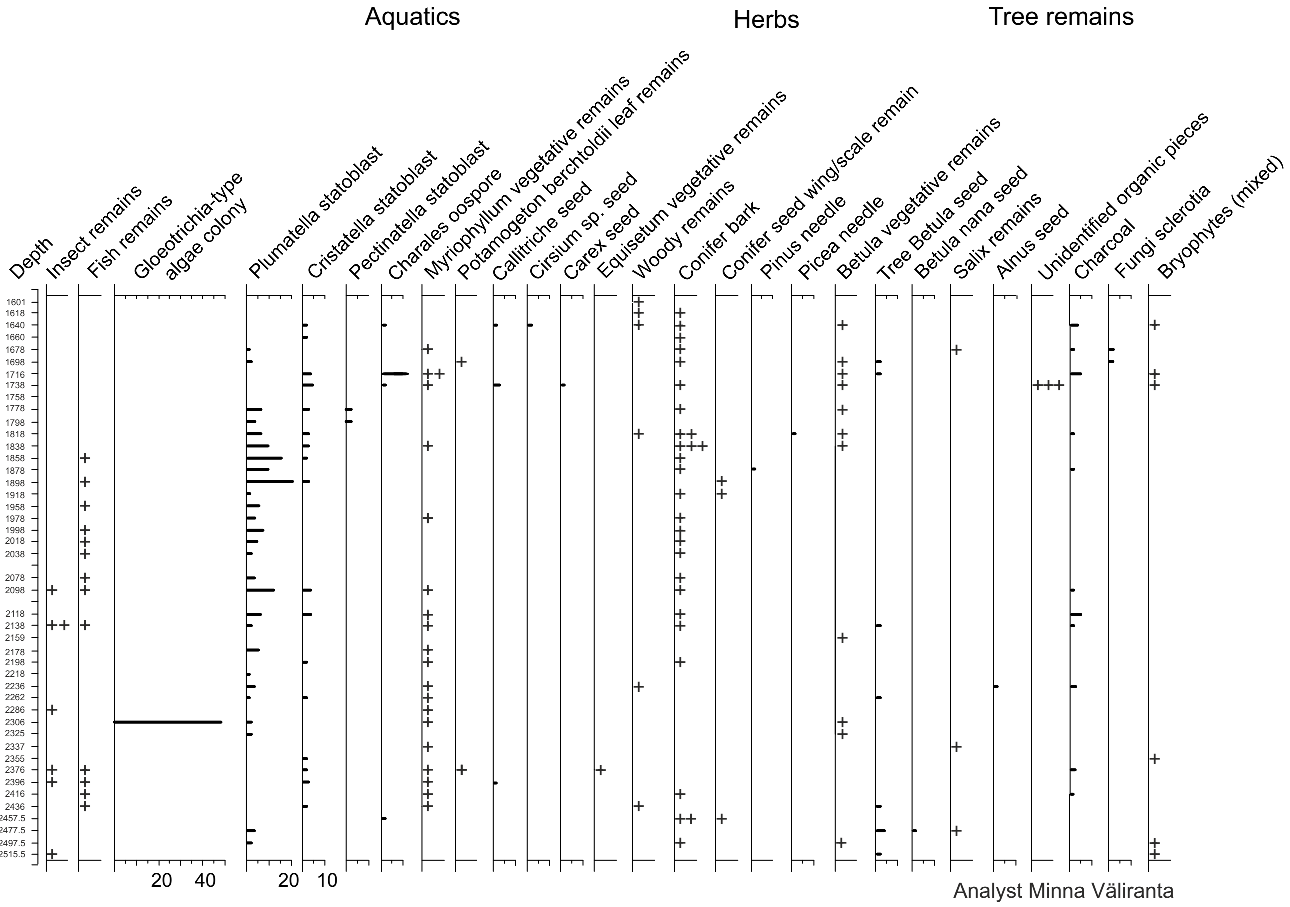

Supplementary Figure 2. Full macrofossil data for the Sokli sequence. Macrofossil types that can be individually counted are indicated with bars. Others are expressed using a qualitative estimate of relative abundance (+ present, ++ frequent, +++ abundant).

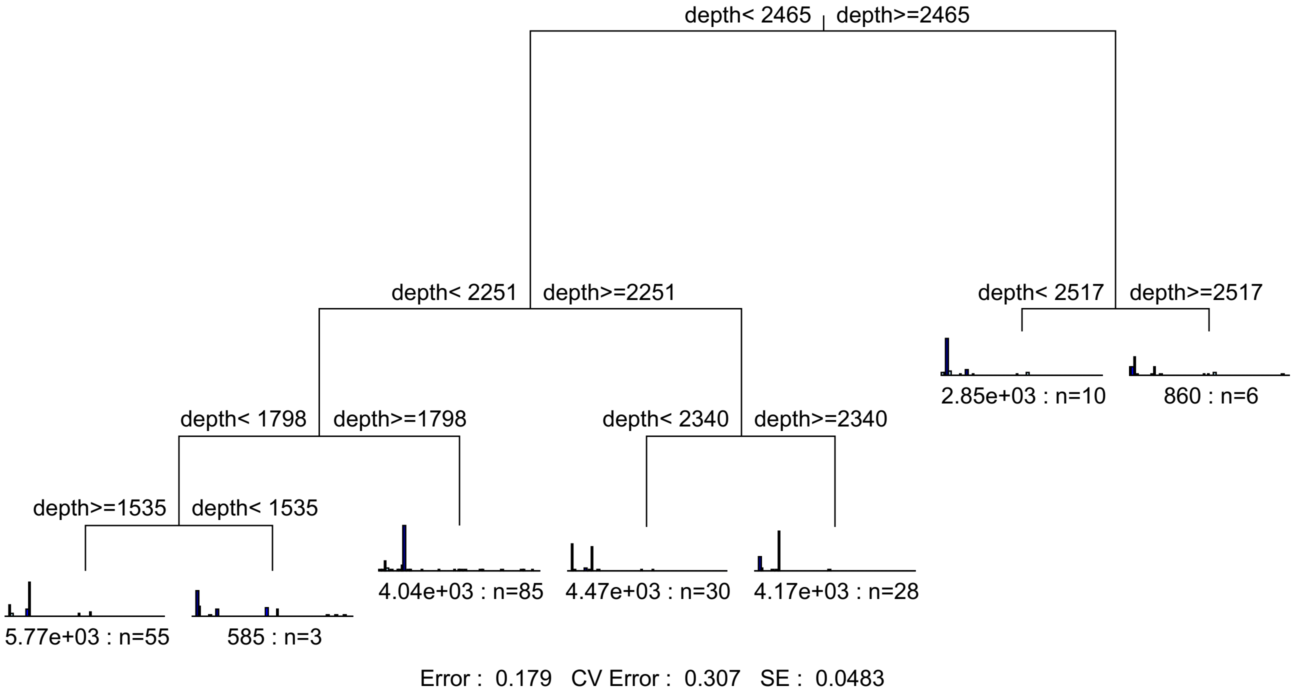

Supplementary Figure 3. Multivariate regression tree of the Sokli Eemian pollen sequence.

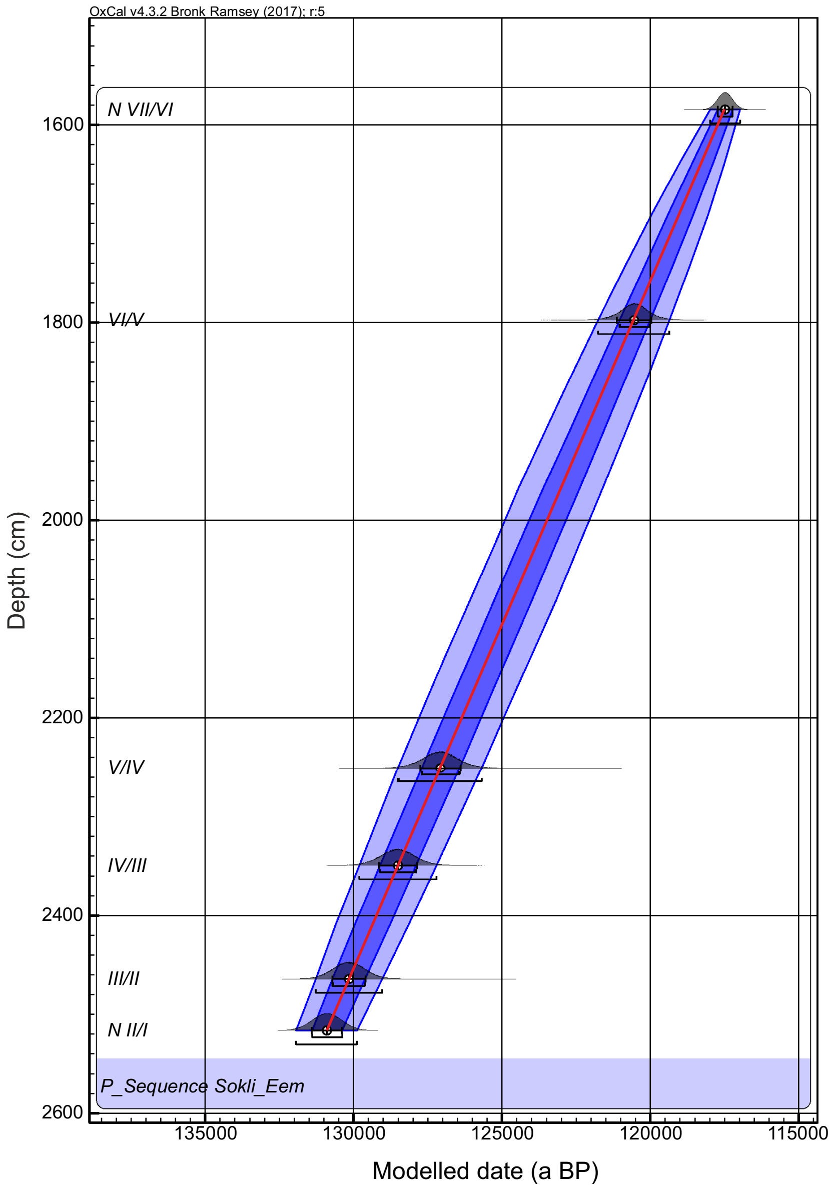

Supplementary Figure 4. Age-depth model for the Sokli Eemian sequence constructed using OxCal 4.3[1]. Chronological uncertainty is estimated based on the spread of the Markov Chain Monte Carlo runs. The model is constrained by two chronological tie points and associated uncertainty at the base and top of the sequence. Shown are the median (red line) as well as the 68.2% (dark blue) and 95.4% (light blue) confidence envelopes. The modeled chronological probability density functions for each pollen zone transition is shown. For further details see Methods.

Supplementary Table 1. Parameterization and cross-validation performance of the used pollen–climate calibration models. Models were created between surface pollen samples and two climate parameters: mean July temperature (T_{jul}) and mean January temperature (T_{jan}). Six calibration methods were used: weighted averaging (WA), weighted averaging partial least squares (WA-PLS), maximum likelihood response surfaces (MLRC), the modern analogue technique (MAT), random forest (RF) and the boosted regression tree (BRT). The BRT models use four parameters: learning rate (LR), maximum number of trees (MT), tree complexity (TC), and bagging fraction (BF). Model performance is summarized by two values, the root-mean-square error of prediction (RMSEP) and the maximum (max.) bias, calculated based on residuals in 10-fold cross-validation with the modern calibration data. To assess model performance, the climate gradient covered by the calibration data is also shown. The RMSEP represents a typical prediction error for all samples. Max. bias is calculated as the largest mean of prediction residuals found for any of the 10 equal-length segments of the calibration data climate gradient, and represent a "worst case" prediction error for a specific environment.

Climate parameter	Method	Model parameters	Calibration data gradient (^{o}C)	RMSEP (^{o}C)	Max. bias (^{o}C)
T_{jul}	WA	Monotonic deshrinking, tolerance downweighting	5.6–24.4	1.68	6.88
T_{jul}	WA-PLS	2 components	5.6–24.4	1.80	6.46
T_{jul}	MLRC	Default	5.6–24.4	2.05	5.22
T_{jul}	MAT	Weighted mean of 5 analogues, squared chord distance	5.6–24.4	1.12	8.27
T_{jul}	BRT	LR=0.025, MT=3000, TC=4, BF=0.5	5.6–24.4	1.15	3.31
T_{jul}	RF	Number of trees = 100	5.6–24.4	1.25	7.63
T_{jan}	WA	Monotonic deshrinking, tolerance downweighting	−25.1–6.2	3.13	13.26
T_{jan}	WA-PLS	3 components	−25.1–6.2	3.19	9.77
T_{jan}	MLRC	Default	−25.1–6.2	3.44	5.97
T_{jan}	MAT	Weighted mean of 5 analogues, squared chord distance	−25.1–6.2	1.79	3.20
T_{jan}	BRT	LR=0.025, MT=3000, TC=4, BF=0.5	−25.1–6.2	1.99	5.01
T_{jan}	RF	Number of trees = 100	−25.1–6.2	2.17	8.32

Supplementary Table 2. Taxon response metrics for the pollen–climate calibration models for July mean temperature (T_{jul}). The taxa are ranked according to their relative importance in the boosted regression tree (BRT) calibration model. The BRT relative importance is expressed as %, calculated based on the number of times the taxon is used in tree splits and the associated improvement seen in model performance. Modern prevalence (%) and fossil prevalence (%) indicate the portion of calibration or fossil (respectively) samples the taxon is recorded in. The fossil prevalence together with the BRT relative importance roughly indicate how much the taxon is likely to affect the palaeoclimate reconstruction, i.e., how often the species is found in the fossil data, and if found, how much it likely influences the reconstruction. The climate indicator values of the taxa in the calibration models are summarized by the BRT partial dependence plot, showing the individual response to the taxon in the BRT model (taxon abundance on x axis, climate response on y axis) and by the unimodal optimum and tolerance of the taxon in the weighted averaging (WA) model.

Relative importance rank (BRT)	Taxon	Relative importance % (BRT)	Modern prevalence (%)	Fossil prevalence (%)	WA response (optimum ± tolerance, °C)	BRT partial dependence plot
1	Selaginella	20.7	19.2	5.5	10.4 ± 1.4	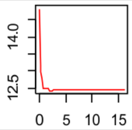
2	Cyperaceae	7.7	95.9	100.0	13.5 ± 3.3	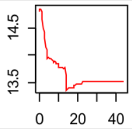
3	Quercus (deciduous)	6.1	65.9	18.4	16.9 ± 2.4	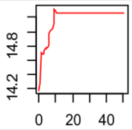
4	Alnus	5.9	98.5	100.0	15.8 ± 2.3	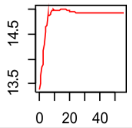
5	Artemisia	5.4	72.4	33.6	16.4 ± 2.7	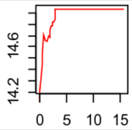
6	Juniperus	4.9	77.9	81.6	12.6 ± 2.4	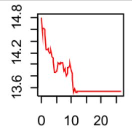
7	Betula	4.0	99.1	100.0	14 ± 2.6	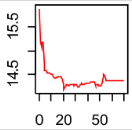
8	Chenopod-iaceae	3.9	55.5	15.7	17.4 ± 2.9	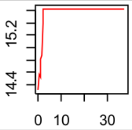
9	Polypod-iaceae	3.7	90.7	71.4	12.4 ± 2.3	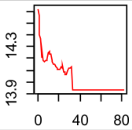
10	Lycopod-iaceae	3.2	50.6	69.6	12.2 ± 2.4	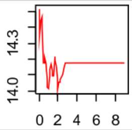

Supplementary Table 3. Taxon response metrics for the pollen–climate calibration models for January mean temperature (T_{jan}). For explanation see caption to Table S2.

Relative importance rank (BRT)	Taxon	Relative importance % (BRT)	Modern prevalence (%)	Fossil prevalence (%)	WA response (optimum ± tolerance, °C)	BRT partial dependence plot
1	Quercus (deciduous)	14.8	65.9	18.4	−1.7 ± 3.3	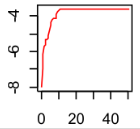
2	Picea	13.2	91.9	98.6	−9 ± 4.8	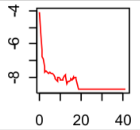
3	Corylus	12.7	73.0	40.6	−1.8 ± 4.1	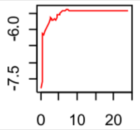
4	Betula	12.5	99.1	100.0	−8.7 ± 5.6	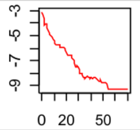
5	Poaceae	8.8	99.6	97.2	−4.3 ± 5	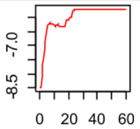
6	Rumex / Oxyria	4.0	87.6	2.3	−4.7 ± 4.1	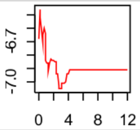
7	Pinus	4.0	99.9	100.0	−7.9 ± 5.2	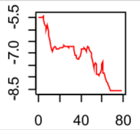
8	Ericaceae	3.1	83.6	49.3	−2.5 ± 5.9	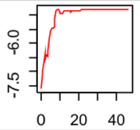
9	Polypod-iaceae	2.0	90.7	71.4	−5 ± 4.5	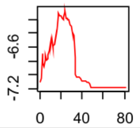
10	Artemisia	2.0	72.4	33.6	−7.5 ± 6.1	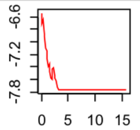

Supplementary Methods

Climate reconstruction methods

Calibration data. We synthesized a new pollen–climate calibration dataset based on the European Modern Pollen Database[2] (EMPD). We selected all EMPD lake samples but excluded samples from semiarid and dry-summer climates, to focus the pollen–climate modelling on taxa which are likely components in the northern-European vegetation during the Eemian. The final dataset includes 807 lakes. The EMPD species data for the selected samples were synthesized into 73 terrestrial pollen and spore types. Climate data for the pollen samples were extracted from CRU CL v. 2.0 climate grids[3] with the RASTER library[4] for R, using bilinear interpolation based on four closest grid cells and lapse-rate corrected (6.4°C/km) based on the difference between site elevation and grid cell elevation.

Climate variables. Eemian palaeoclimate reconstructions were prepared for mean July temperature (T_{jul}) and mean January temperature (T_{jan}). These variables were chosen due to their ecological influence on vegetation[5–7] and their low correlation (r = 0.27) in the calibration data, which facilitates the modelling of the independent effect of each variable[8]. A third, potentially ecologically influential variable, water balance (WAB), was not included due to a smaller independent influence in partial CCA's run with the calibration data (implemented in VEGAN version 2.2-0[9]), with WAB having 1.9 % independent influence (T_{jul} and T_{jan} as covariables) and T_{jan} 4.8 % independent influence (T_{jul} and WAB as covariables). It is likely that T_{jan} and WAB have generally co-varied in the past, with both increasing with the incursion of Atlantic low-pressure systems, making the choice between these variables non-critical.

Calibration methods. Six different calibration methods were used to create pollen–climate models: weighted averaging[10] (WA) and weighted averaging-partial least squares[11] (WA-PLS) regression, maximum likelihood response surfaces[10] (MLRC), the modern analogue technique[12] (MAT), and two machine-learning approaches based on regression tree ensembles, the random forest[13] (RF) and the boosted regression tree[14] (BRT). The reconstruction algorithms were implemented in R (version 3.0.3)[15], using the libraries GBM (version 2.1)[16], RANDOMFOREST (version 4.6-10)[17], and RIOJA (version 0.8-7)[18]. For further details about the calibration models see Table S1.

Validation of climate reconstructions. Reconstruction errors, estimated by 10-fold cross-validations, are in the range of 1.12–2.05°C for T_{jul} and 1.79–3.44°C for T_{jan}, depending on model (Table S1). Although the model performance was assessed by modern cross-validation, there is a possible further error component in palaeoreconstruction due to fossil samples lacking good modern analogues in the calibration data[19]. The problem of non-analogue samples in palaeo-reconstruction is acknowledged in literature, however the sensitivity of individual reconstruction algorithms remains poorly understood. Some recent studies using simulated non-analogue fossil data have suggested that regression-tree ensemble models (here, RF and BRT) may be more robust in non-analogue conditions compared to classical methods such as WA, WA-PLS, and MAT[20,21]. Due to these challenges in model evaluation,

we use all six methods as they all showed acceptable performance in cross-validations, as well as generally similar and realistic reconstructed palaeoclimate patterns. To estimate the likelihood of non-analogue errors, we estimated for each fossil pollen sample the compositional distance (squared chord distance) to the closest analogue in the modern pollen data. To assess the Eemian analogue distances, we also calculated the mean modern analogue distance for late-Holocene samples from a nearby lake core[22].

As a further validation step, we test the statistical significance of the palaeoclimate reconstructions using a test[23] implemented in the PALAEOSIG library (version 1.1-2)[24] for R. This test uses redundancy analysis to assess whether the palaeo-reconstruction explains more fossil data variation compared to reconstructions of 999 random variables. Testing with reconstructions prepared from simulated fossil data[25] have suggested that this significance test is more reliable in identifying robust reconstructions compared to cross-validations run with the modern calibration data. The significance tests were run for the MAT reconstruction, as the other methods may be susceptible to false negatives with fossil data characterized by a small effective number of species[22], and this is the case with our Eemian dataset (Hill's N2 = 2.87). Given the similar patterns in reconstructed T_{jul} and T_{jan} with most methods (Fig. 3A,B), the significance of the MAT reconstruction is taken as representative for the multi-method median. In implementing the test, we first tested the entire fossil sequence. This produced a significant result for T_{jul} (p = 0.001) but not for T_{jan} (p = 0.456). Following this, we ran the significant test for T_{jan} by incrementally leaving out pollen zones from both ends of the sequence, to identify the longest continuous segment of the fossil record to produce a significant reconstruction. A significant result (p = 0.011) was found for pollen zones V and VI, including 140 out of 217 fossil pollen samples and spanning the mid-to-late Eemian.

Some recent literature has highlighted the challenges in reconstructing so-called secondary variables, with a comparatively small ecological effect[8,23,25]. Such secondary variables are typically correlated with the primary variable in the used calibration data. Using reconstructions from simulated fossil data[8,25], it is suggested that these calibration-data correlations may induce spurious reflections of the past variability of the primary variable in the palaeo-reconstruction for the secondary variable. In the present work, these challenges are particularly relevant for the presented T_{jan} reconstruction, as analyses of northern European surface pollen data[4] show summer temperature to be the dominant ecological driver. However, literature on northern European modern vegetation[6,7,26] and surface pollen datasets[5] suggest a signal of winter temperature in a subset of taxa or pollen types, and thus an ecological case for T_{jan} reconstruction exists. The structure of the prepared models, as analysed by the most important used predictors (taxa) in BRT's (Tables S2 and S3), is consistent with this ecological knowledge. The relative contribution to the T_{jul} model is distributed between a variety of taxa, while the T_{jan} model is more selective in the use of taxa and includes the well-understood winter indicators *Corylus* and *Quercus*[5,7,26] among the three most important predictors. In the palaeo-reconstructions for T_{jul} and T_{jan}, the first-order trends in the reconstructions are *opposite* (falling T_{jul}, rising T_{jan}; Fig. 3) and thus the trend in T_{jan} is unlikely to be driven by the weak *positive* correlation with T_{jul} (r = 0.27) in the calibration data. The first-order trend of increasing T_{jan} is supported by independent diatom data[27] and the statistical significance testing[23].

Supplementary References

1. Bronk Ramsey, C. Bayesian analysis of radiocarbon dates. *Radiocarbon* **51**, 337–360 (2009).
2. Davis, B.A.S. et al. The European Modern Pollen Database (EMPD) project. *Veget. Hist. Archaeobot.* **22**, 521–530 (2013).
3. New, M., Lister, D., Hulme, M. & Makin, I. A high-resolution data set of surface climate over global land areas. *Clim. Res.* **21**, 1–25 (2002).
4. Hijmans, R.J. *raster: Geographic data analysis and modeling. R package version 2.2-31.* http://CRAN.R-project.org/package=raster (2002).
5. Salonen, J.S., Seppä, H., Luoto, M., Bjune, A. & Birks, H.J.B. A North European pollen–climate calibration set: analysing the climate response of a biological proxy using novel regression tree methods. *Quat. Sci. Rev.* **45**, 95–110 (2012).
6. Skov, F. & Svenning, J.-C. Potential impact of climatic change on the distribution of forest herbs in Europe. *Ecography* **27**, 366–380 (2004).
7. Sykes, M.T., Prentice, I.C. & Cramer, W. A bioclimatic model for the potential distributions of north European tree species under present and future climates. *J. Biogeogr.* **23**, 203–233 (1996).
8. Juggins, S. Quantitative reconstructions in palaeolimnology: New paradigm or sick science? *Quat. Sci. Rev.* **64**, 20–32 (2013).
9. Oksanen, J. et al. *vegan: Community Ecology Package. R package version 2.2-0.* http://CRAN.R-project.org/package=vegan (2014).
10. Birks, H.J.B., ter Braak, C.J.F., Line, J.M., Juggins, S. & Stevenson, A.C. Diatoms and pH reconstruction. *Philos. Trans. Royal Soc. B* **327**, 263–278 (1990).
11. ter Braak, C.J.F. & Juggins, S. Weighted averaging partial least squares regression (WA-PLS): An improved method for reconstructing environmental variables from species assemblages. *Hydrobiologia* **269–270**, 485–502 (1993).
12. Overpeck, J.T., Webb III, T. & Prentice, I.C. Quantitative interpretation of fossil pollen spectra: dissimilarity coefficients and the method of modern analogs. *Quat. Res.* **23**, 87–108 (1985).
13. Breiman, L. Random forests. *Mach. Learn.* **45**, 5–32 (2001).
14. De'ath, G. Boosted trees for ecological modeling and prediction. *Ecology* **88**, 243–251 (2007).
15. R Core Team. *R: A language and environment for statistical computing.* R Foundation for Statistical Computing, Vienna, Austria. http://www.R-project.org/ (2014).
16. Ridgeway, G. *gbm: Generalized boosted regression models. R package version 2.1.* http://CRAN.R-project.org/package=gbm (2013).
17. Liaw, A. & Wiener, M. Classification and Regression by randomForest. *R News* **2(3)**, 18–22 (2002).
18. Juggins, S. *rioja: Analysis of Quaternary Science Data, R package version (0.8-7).* http://CRAN.R-project.org/package=rioja (2012*).*
19. Jackson, S.T. & Williams, J.W. Modern Analogs in Quaternary Paleoecology: Here Today, Gone Yesterday, Gone Tomorrow? *Annu. Rev. Earth Planet. Sci.* **32**, 495–537 (2004).

20. Goring, S., Salonen, J.S., Luoto, M. & Williams, J. Non-analogues in paleoecological reconstruction: model behaviour and implications. In: Dy, J.G., Emile-Geay, J., Lakshmanan, V. & Liu, Y. (Eds.), *Proceedings of the Fifth International Workshop on Climate Informatics: CI 2015* (2015).
21. Juggins, S., Simpson, G.L. & Telford, R.J. Taxon selection using statistical learning techniques to improve transfer function prediction. *Holocene* **25**, 130–136 (2015).
22. Salonen, J.S., Helmens, K.F., Seppä, H. & Birks, H.J.B. Pollen-based palaeoclimate reconstructions over long glacial–interglacial timescales: methodological tests based on the Holocene and MIS 5d–c deposits at Sokli, northern Finland. *J. Quat. Sci.* **28**, 271–282 (2013).
23. Telford, R.J. & Birks, H.J.B. A novel method for assessing the statistical significance of quantitative reconstructions inferred from biotic assemblages. *Quat. Sci. Rev.* **30**, 1272–1278 (2011).
24. Telford, R.J. *palaeoSig: Significance Tests of Quantitative Palaeoenvironmental reconstructions. R package version 1.1-2.* http://CRAN.R-project.org/package=palaeoSig (2013).
25. Rehfeld, K., Trachsel, M., Telford, R.J. & Laepple, T. Assessing performance and seasonal bias of pollen-based climate reconstructions in a perfect model world. *Clim. Past* **12**, 2255–2270 (2016).
26. Seppä, H. et al. Trees tracking a warmer climate: the Holocene range shift of hazel (Corylus avellana) in northern Europe. *The Holocene* **25**, 53–63 (2015).
27. Plikk, A. et al. Development of an Eemian (MIS 5e) Interglacial palaeolake at Sokli (N Finland) inferred using multiple proxies. *Palaeogeogr. Palaeoclimatol. Palaeoecol.* **463**, 11–26 (2016).
