# Supplementary material for: Abrupt high-latitude climate events and decoupled seasonal trends during the Eemian
Source: Nat Commun. 2018 Jul 20;9:2851. doi: 10.1038/s41467-018-05314-1 (PMC6054633; doi:10.1038/s41467-018-05314-1)
Supplement: Supplementary file 3 — Description of Additional Supplementary Files [file 41467_2018_5314_MOESM3_ESM.pdf]

Description of Additional Supplementary Files

File Name: Supplementary Data 1
Description: Palaeoclimate reconstructions, fossil data, reconstruction model calibration data.

File Name: Supplementary Data 2
Description: R code for preparing the palaeoclimate reconstructions.
